# Oromotor variability in children with mild spastic cerebral palsy: a kinematic study of speech motor control

**DOI:** 10.1186/1743-0003-7-54

**Published:** 2010-10-27

**Authors:** Chia-ling Chen, Hsieh-ching Chen, Wei-hsien Hong, Fan-pei Gloria Yang, Liang-yi Yang, Ching-yi Wu

**Affiliations:** 1 Department of Physical Medicine and Rehabilitation, Chang Gung Memorial hospital, 5 Fuhsing St. Kweishan, Taoyuan 33302, Taiwan; 2 Graduate Institute of Early Intervention, Chang Gung University, 259 Wenhwa 1 Rd., Kweishan, Taoyuan 33302, Taiwan; 3 Department of Industrial Engineering and Management, Chaoyang University of Technology, 168 Jifong E. Rd., Wufong, Taichung County 41349, Taiwan; 4 Department of Sports Medicine, China Medical University, 91 Hsueh-Shih Rd., Taichung, 40402, Taiwan; 5 Department of Radiology and Biomedical Imaging, University of California at San Francisco, 185 Berry Street Suite 350, San Francisco, CA 94107, USA; 6 Department of Occupational Therapy, Chang Gung University, 259 Wenhwa 1 Rd., Kweishan, Taoyuan 33302, Taiwan

## Abstract

**Background:**

Treating motor speech dysfunction in children with CP requires an understanding of the mechanism underlying speech motor control. However, there is a lack of literature in quantitative measures of motor control, which may potentially characterize the nature of the speech impairments in these children. This study investigated speech motor control in children with cerebral palsy (CP) using kinematic analysis.

**Methods:**

We collected 10 children with mild spastic CP, aged 4.8 to 7.5 years, and 10 age-matched children with typical development (TD) from rehabilitation department at a tertiary hospital. All children underwent analysis of percentage of consonants correct (PCC) and kinematic analysis of speech tasks: poly-syllable (PS) and mono-syllable (MS) tasks using the Vicon Motion 370 system integrated with a digital camcorder. Kinematic parameters included spatiotemporal indexes (STIs), and average values and coefficients of variation (CVs) of utterance duration, peak oral opening displacement and velocity. An ANOVA was conducted to determine whether PCC and kinematic data significantly differed between groups.

**Results:**

CP group had relatively lower PCCs (80.0-99.0%) than TD group (*p *= 0.039). CP group had higher STIs in PS speech tasks, but not in MS tasks, than TD group did (*p *= 0.001). The CVs of utterance duration for MS and PS tasks of children with CP were at least three times as large as those of TD children (*p *< 0.01). However, average values of utterance duration, peak oral opening displacement and velocity and CVs of other kinematic data for both tasks did not significantly differ between two groups.

**Conclusion:**

High STI values and high variability on utterance durations in children with CP reflect deficits in relative spatial and/or especially temporal control for speech in the CP participants compared to the TD participants. Children with mild spastic CP may have more difficulty in processing increased articulatory demands and resulted in greater oromotor variability than normal children. The kinematic data such as STIs can be used as indices for detection of speech motor control impairments in children with mild CP and assessment of the effectiveness in the treatment.

## Background

Cerebral palsy (CP) refers to a group of developmental disorders in movement and posture, which are attributed to non-progressive disturbances that occurred in the developing fetal or infant brain [1]. Disturbed neuromuscular control of speech mechanism often result in communication disorders, especially poor speech production in patients with CP [2]. Impaired speech functions such as articulation disorders are present in 38% children with CP [3]. Reduced intelligibility in children with CP can adversely impact communication abilities and limit their vocational, educational, and social participation [4]. Such limitations may consequently diminish these children's quality of life [4].

Children with spastic CP commonly exhibit dysarthria of varying severities. One of the primary characteristics of dysarthria is articulatory imprecision [5]. Some fairly stable features of CP dysarthria include inaccurate articulatory place and manner of consonants [6]. Specifically, at the phonemic level, patients with dysarthria display anterior lingual place inaccuracy, reduced precision of fricative and affricate manners, and inability to achieve the extreme positions in the vowel articulatory space [6]. In addition, previous studies revealed that speakers with CP exhibit smaller vowel working space areas compared to age-matched controls and that the width of vowel working space area significantly correlates with vowel and word intelligibility [7].

Quantitative measurements of speech motor control have been used to characterize language and communication deficits in diverse patient populations except patients with CP. These measurements include kinematic [8-11], kinetic [12], electromyographic (EMG) [12-16] and acoustic analyses [17-19]. Kinematic measures of articulatory movements include measurements of movement amplitude, velocity and duration [11], and speech movement trajectory analysis [10,11]. The spatio-temporal index (STI) values in speech movement trajectory analysis reflect the degree to which repeated performance of a task produces movement trajectories that converge on a single pattern [10]. Therefore, the STI values indicate the degree of oromotor stability of a speech task that produces movement trajectories [10]. At present, lip and jaw kinematic analyses in previous studies have identified the speech motor control pattern in children with normal development [9,12,20,21]. However, no studies up to date have performed kinematic analysis of speech motor control in children with mild spastic CP.

It is important to conduct speech motor control analysis in children with CP for several reasons. First, quantitative measures of motor control are considerably more sensitive than conventional methods in determining the distribution and nature of orofacial motor impairments which degrade fine motor performance [22]. A research has reported that the most frequent abnormalities of subjects with athetoid CP included large ranges of jaw movement, inappropriate positioning of the tongue for various phonetic segments, intermittency of velopharyngeal closure caused by an instability of velar elevation, prolonged transition times for articulatory movements, and retrusion of the lower lip [23]. The kinematic analysis will provide quantified indices to characterize the abnormalities described by the conventional analysis.

Secondly, treating motor speech dysfunction in children with CP requires an understanding of the mechanism underlying speech motor control. Previous research has demonstrated that the measures of dynamics in select structures of the oral motor system were found to be related to impairments in speech intelligibility [22]. Even in mild CP patients with intelligence levels above 70, half of the patients exhibit motor speech problems [2]. However, it remained unclear how the fine articulator movements are controlled and coordinated for speech production in children with mild spastic CP. Understanding the control and coordination mechanism for speech production is essential for developing appropriate treatment.

We hypothesize that speech motor control is impaired in children with mild spastic CP because these children have greater oromotor variability than TD children. We predict that CP children's oromotor variability can be reflected in high variability on kinematic variables and high STI values in speech tasks. This study aims to investigate speech motor control in children with mild spastic CP using kinematic analysis. The kinematic parameters used to detect speech motor control problems in the present study may potentially have practical clinical applications.

## Methods

### Participants

Ten children with mild spastic CP (seven male, three female), aged 4.8 to 7.5 years old (mean age: 5.9 ± 1.0 years), from rehabilitation department at a tertiary hospital, Chang Gung Memorial hospital, were enrolled in the study. The inclusion criteria were as follows: (1) mild spastic CP with Gross Motor Functional Classification System (GMFCS) [24] levels I-II; (2) ability to perform speech tasks with mild articulation disorders; (3) good cooperation during examination; and (4) ability to understand the verbal commands required for analysis. The GMFCS grades the self-initiated movement of CP patients with particular emphasis on their functional abilities (sitting, crawling, standing and walking) and their need for assistive devices (e.g., walkers, crutches canes and wheelchairs). The GMFCS employs a 5-point scale (I-V) from "independent" (level I) to "dependent: (level V). Four children with CP were at GMFCS level I, and six children with CP were at level II. Exclusion criteria were any history of the following conditions within the previous three months: (1) significant medical problems such as active pneumonia or urinary tract infection; (2) significant hearing impairment; (3) any major surgical treatment such as orthopedic surgery or neurosurgical surgery; (4) any treatment with nerve or motor point block such as a botulinum toxin injection; and (5) history of facial palsy.

The control group consisted of ten age-matched children with typical development (TD) (six male and four female) aged 4.9 to 7.5 years (mean age: 6.1 ± 0.8 years) with no history of learning disabilities, speech impairment, such as specific speech production errors, language impairments, neurological lesions, or visual or hearing impairment. The speech functions were screened by a speech pathologist. The institutional review board for human studies at Chang Gung Memorial hospital approved the study protocol. All participants and their parents or guardians provided informed consent to participate in the study.

### Instrumentation

Kinematic analysis of head and mouth movements during speech tasks was performed using the Vicon Motion 370 system (Oxford metrics Ltd, UK) integrated with a digital camcorder. The Vicon system, which consisted of six infrared cameras, was used in conjunction with a personal computer to capture the movement of reflective markers. Kinematic data for the reflective markers were recorded at a sampling rate of 60 Hz and digitally low-pass filtered using a second-order Butterworth filter with 5 Hz cut-off frequency. The 5-Hz cut-off frequency was used to reduce markers' velocity error, which might be introduced by noise signal using numerical differentiation method, without significantly altering the results of marker displacements. For each speech task, a digital signal synchronized with an external LED light was collected by the Vicon system to synchronize the video images and to determine onset and offset of marker movement.

### Assessment Procedures

We analyzed specific speech production errors, speech intelligibility and performed kinematic analysis of speech tasks on all children. In addition to these analyses, we also analyzed motor severity of children with CP. The speech pathologist who screened patients' speech functions assessed each patient's specific speech production errors. A physiatrist (CL Chen) classified the motor severity of CP in each child using GMFCS [24]. Demographic data of all participants, including age and gender were recorded. Demographic data did not significantly differ between children with CP and children with TD.

#### Experimental setup for measuring speech intelligibility

Each child was seated in a quiet room. The recording system used to measure speech intelligibility consisted of an external microphone and a laptop computer (IBM ThinkPad 570E) with 16 k Hz sampling rate and 16-bit resolution. The microphone was placed on a table approximately 15 cm from the mouth of the child. The children were shown pictures or texts printed on cards and asked to read them aloud in a normal voice. Whenever the child encountered an unfamiliar word, the examiner explained the word or asked the child to read it with the assistance of phonetic transcription. The examiner did not model the correct sound production or provide other assistance. The speech recording tasks included 69 picture-cards for preschool children and 140 word-cards for school children. Before all speech tasks started, the examiner told the subjects that the words they read were going to be recorded. The examiner recorded a speech sample of each subject for each speech task.

The percentage of consonants correct (PCC), modified from procedures outlined by Shriberg and Kwiatkowski (1982), was used to determine severity of speech intelligibility [25]. The PCC information was used as an index to quantify severity of involvement [25]. To measure PCC, a rater must make correct-incorrect judgments of individual sounds produced in the speech sample of each subject. The same rater, who was a native Mandarin speaker with normal hearing, transcribed recorded speech samples. The PCC was calculated as 100 × (number of correct consonants/number of correct plus incorrect consonants) [25]. The PCC ranged from 80.0-99.0% in children with CP, and 95.5-100.0% in TD children. In order to test intra-rater and inter-rater reliabilities, a research assistant was recruited to rate the sound of 10 children, half from CP groups and half from TD group, randomly selected from the data base. The intra-class correlation coefficient (ICC) values of inter-rater and intra-rater reliability for PCC were 0.812 and 0.977, respectively.

Additionally, the same speech pathologist identified all subjects' specific speech production errors based on the phonological process analysis [26] from the recorded speech samples. The patterns of phonological process analysis consisted of assimilation, fronting, backing, stopping, voicing, de-voicing, affrication, de-affrication, nasalization, de-nasalization, and lateralization [26]. Five children had specific speech production errors: stopping and voicing (2 cases), backing (one case), fronting and de-affrication (one case), and other error (one case).

#### Experimental setup of Kinematic analysis

**Figure 1:**
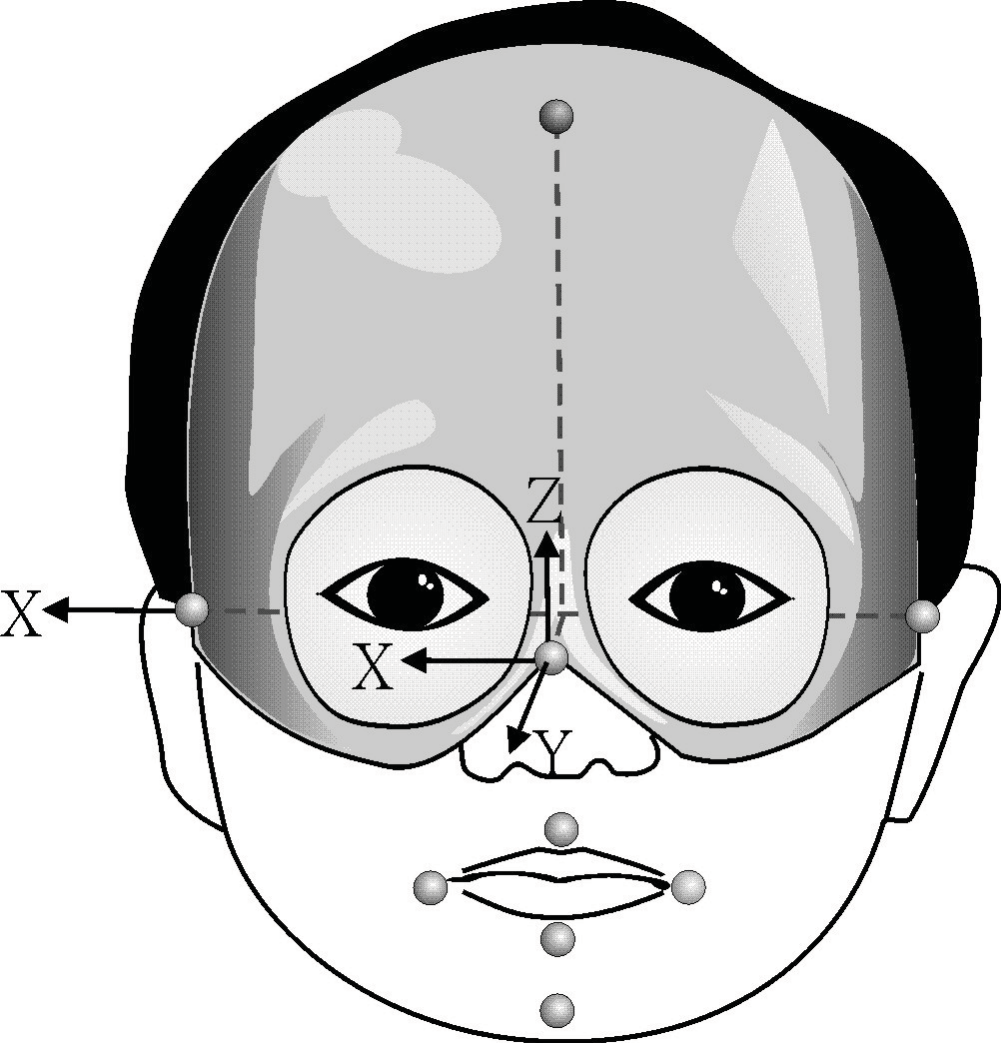
**Experimental setup for kinematic analysis of speech tasks**. The reflective markers were attached to the facial mask and oral areas and reference coordination system was established by marks on the mask.

During the Kinematic analysis task, the subjects were comfortably seated in chairs adjusted to 100% of lower leg length, measured from the lateral knee joint to the floor with the subject standing. The trunk was secured to the chair-back with a harness in order to minimize trunk flexion and rotation. Each subject wore a plastic facial mask with an adjustable set of elastic belts to keep it skin-tight and to help the mask eyelets fit in the subject's eye sockets (Figure 1A). Four reflective markers in diameter of 0.6 cm were attached to the facial mask at the forehead, bilateral pre-auricular areas and nose to establish a reference coordination system with the positive x, y, z orientation line in horizontal rightward, anterior-posterior, and vertical upward directions respectively (Figure 1B). The direction of *x*-axis is defined along the line joining the bilateral markers at per-auricular areas. The *y*-axis is perpendicular to the frontal plane passing through markers at the forehead and bilateral pre-auricular areas. The *z*-axis is orthogonal to *x*- and *y*-axis. The origin of reference coordination system was located at the nose marker. The use of mask helps to establish a reliable coordinate system of head and to minimize artificial error caused by movement of facial skin. For oral-movement tracking, five markers were attached to the bilateral mouth corners, the upper and lower lips at midline and jaw region (Figure 1A). That is, nine reflective markers were used on the facial mask and oral areas (Figure 1C).

All participants underwent mono-syllable (MS) and poly-syllable (PS) task assessments. The stimuli of the speech tasks were consonant-vowel syllables. Each syllable consisted of one of the two bilabial consonants (/p/,/p^h^/) and one of the five basic vowels (/a/,/i/,/u/,/æ/, and/o/). These vowels are selected because they are the most common in human languages [27]. Among these vowels,/a/,/i/, and/u/are most commonly used in Mandarin language [7], the native language spoken by the subjects. We chose the bilabial consonants to elicit the lip opening-closing movement in each consonant-vowel syllable. For both tasks, the examiner pronounced the syllables themselves and asked participants to repeat after the examiner. The examiner said the target syllable(s) at a relatively slow rate for clarity purpose. During the MS tasks, participants were asked to speak/pa/,/pi/,/p^h^u/,/p^h^æ/, and/p^h^o/separately. During the PS task, participants were required to speak/pa, pi, p^h^u, p^h^æ, p^h^o/ in a sequence.

The order of task presentation was randomized. Each task was repeated at least 10 times until we collected ten usable trials for each task in each individual. If markers' kinematic data were not correctly captured, these trials were excluded and retested. We used ten trials in each task for analysis. All participants were allowed a 5-sec rest period between each trial repetition and a 15-sec rest period between each task. All participants were allowed three practice trials to familiarize themselves with the experimental setup. A vocal cue together with an LED-light signal was provided to indicate the start of the task by the examiner.

### Data analysis

**Figure 2:**
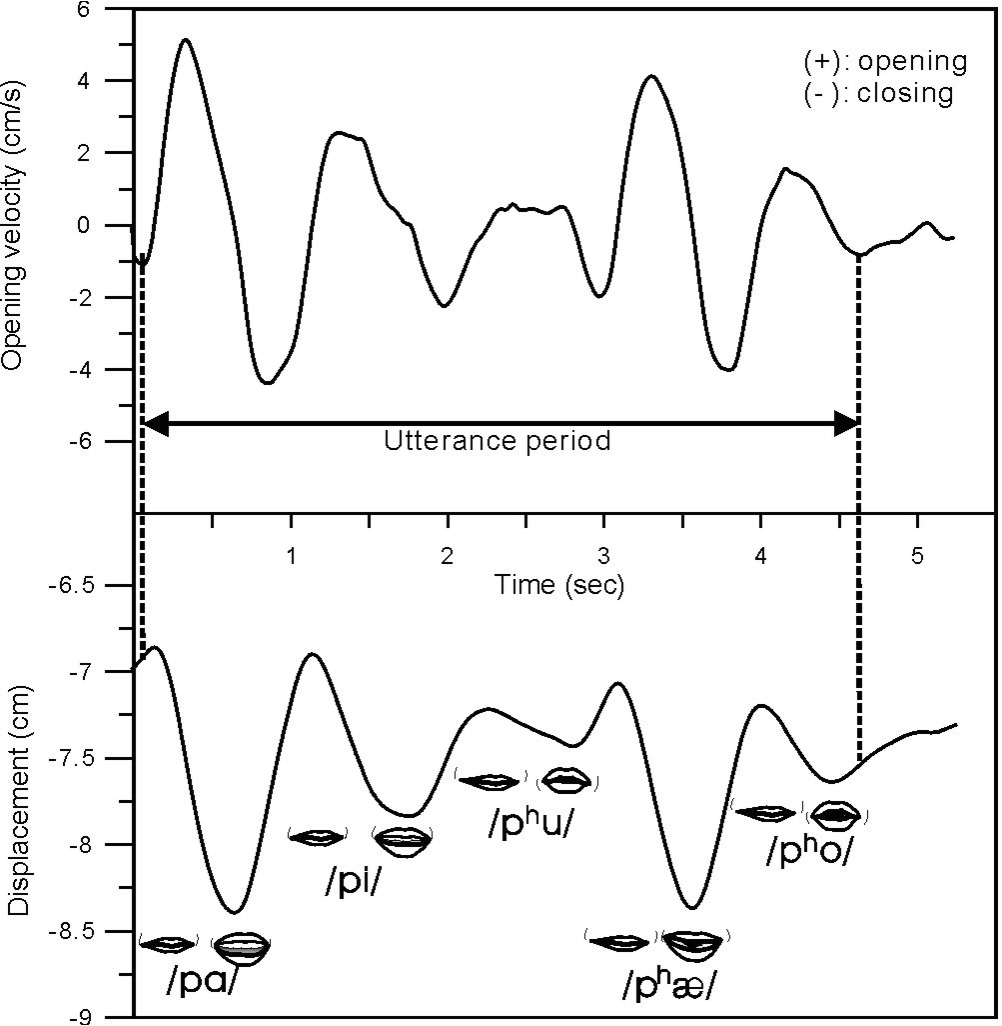
**Illustration indicates the data used in analyses for poly-syllable speech task**. Left vertical line is identified as the instance of peak closing velocity right before the initial opening of the lower lip marker. Right vertical line is defined as the instance when the lower lip was at the peak velocity of its closing movement during the final syllable of the lower lip marker. Both vertical lines mark the displacement period for spatiotemporal index (STI) analysis and the time interval between two points used to measure overall utterance duration in the poly-syllable speech task.

An analysis program for kinematic data coded by LabView (National Instruments, USA) was developed to process the kinematic data. Only the kinematic data of vertical movement (oral aperture in the z-axis) of lip markers were analyzed in this study. The utterance duration, peak oral opening displacement, peak oral opening velocity and STIs of each task were analyzed while performing speech tasks. The overall utterance period of a speech task was determined from the instance of peak closing velocity right before the initial opening of the lower lip to the instance when the lower lip was at the peak velocity of its closing movement during the final syllable (Figure 2). The acoustic traces were used to verify the kinematically-derived onsets. For each task, lower lip displacement waveforms during individual utterance periods were used for STI analysis (Figure 2). Each lower lip displacement waveform was first amplitude normalized by subtracting the individual mean and dividing by the standard deviation and then time normalized to 100% duration. For time normalization, 101 data points were resampled from each amplitude-normalized waveform by a linear interpolation scheme. One standard deviation was then computed every 2% normalized duration across 10 waveforms of each task. There were 50 (from 2% to 100%) standard deviations computed. These standard deviations were then summed to determine the overall STI [10]. In addition to computing STI, for each PS task, peak opening of mouth (oral aperture) was identified by the maximum vertical distance between the upper- and lower-lip markers within the entire utterance duration. Peak oral opening velocity was calculated by determining the maximum time derivatives of the vertical oral opening displacement. The mean peak oral opening velocity and displacement in a PS or a MS task were determined by averaging the maximum opening velocities and displacements, respectively, of each repeated trial.

Furthermore, the coefficient of variation (CV) for kinematic data (utterance duration, peak opening displacement, and peak opening velocity) obtained by dividing the standard deviation of kinematic data by the mean kinematic data. Larger CV indicates higher variability of kinematic data in speech tasks.

### Statistical Analysis

Group differences in age were compared by an independent t test. Gender differences between groups were determined by Fisher's exact test. An ANOVA was conducted to determine whether PCC and kinematic data (values and CVs of utterance duration, peak opening displacement, and peak opening velocity, and STI) significantly differed between groups. The effect size *d *was calculated for each PCC and kinematic data to index the magnitude of the difference in PCC and kinematic data varied between groups [28]. A Cohen's *d *of at least 0.50 represents a large effect; a *d *of at least 0.30 represents a moderate effect, and a *d *of at least 0.10 represents a small effect [29]. Multiple comparisons were performed on the analysis of speech productions in two groups. A *p *value of < 0.01 was considered statistically significant.

## Results

The ANOVA analysis showed that the CP group had relatively lower PCC scores than TD group with moderate effect, though the difference did not achieve significance (*F*_*1,18 *_= 4.962, effect size *d *= 0.465, *p *= 0.039, Table 1).

**Table 1:** Speech intelligibility and spatiotemporal index in children with cerebral palsy and typical development.

Data	Children groups		ANOVA		
		
	Spastic CP (n =10)	TD (n =10)	***F***_***1,18***_	*p *value	Effect size *d*
Speech intelligibility					
Percentage of consonants correct (PCC)	92.6 ± 7.2	97.7 ± 1.5	4.962	0.039	0.465
Spatiotemporal index (STI)					
Mono-syllable task	19.5 ± 5.1	16.5 ± 4.3	1.937	0.181	0.312
Poly-syllable task	30.1 ± 6.9	21.5 ± 2.3	14.093	0.001*	0.663

*p < 0.01.

Values are expressed as mean ± SD.

CP: cerebral palsy; TD: typical development.

PCC: percentage of consonants correct.

**Figure 3:**
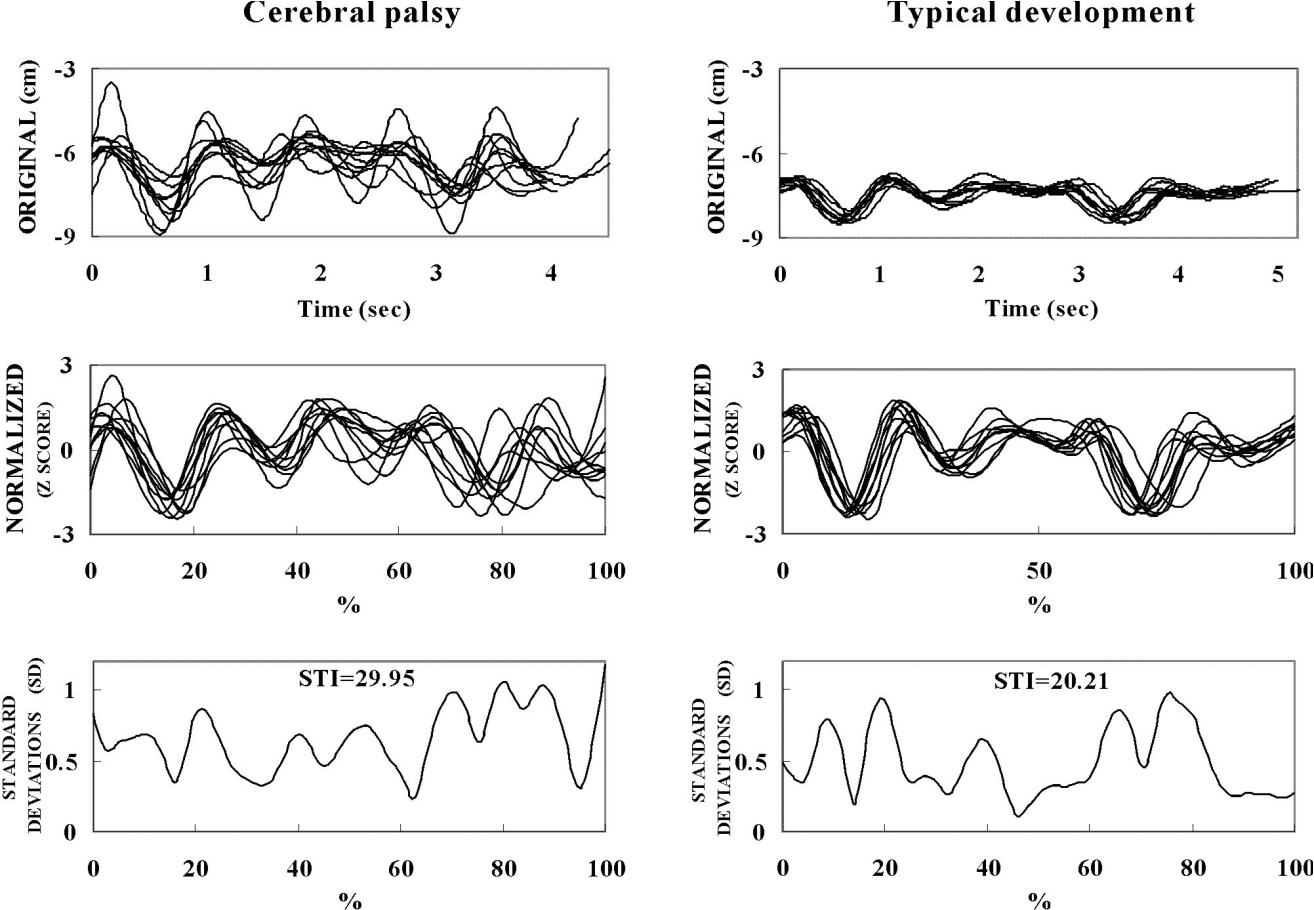
**Illustration indicates the original waveforms, normalized wave forms and spatiotemporal indexes (STIs)**. Comparison of original waveforms, normalized wave forms and STIs between a child with cerebral palsy and a child with typical development.

STI for PS tasks between the CP and TD groups were significantly different (*F*_*1, 18 *_= 14.093, effect size *d *= 0.663, *p *= 0.001, Table 1). However, there were no significant differences in STI of MS tasks between the CP and TD groups (Table 1). The average STI values for PS tasks were greater in CP children than TD children (Table 1). The average STI values of children with mild CP were 19.5 in MS tasks and 30.1 in PS tasks (Table 1). Figure 3 illustrates the original waveforms, normalized waveforms and STIs in PS tasks of one child with CP and one child with TD.

The ANOVA analysis showed no significant differences in the utterance durations, peak oral opening displacement and velocity of both MS and PS tasks between the CP and TD groups (Table 2). The average utterance durations of children with mild CP were 0.95 sec/syllable in both and MS and PS tasks (Table 2). The average peak oral opening displacements of children with mild CP were 1.17 cm in MS tasks and 1.84 cm in PS tasks (Table 2). The average peak oral opening velocities of children with mild CP in MS and PS tasks were 42.4 and 73.5 cm/sec, respectively (Table 2).

**Table 2:** Average values of kinematic data in children with cerebral palsy and typical development.

Kinematic parameters	Children groups		ANOVA		
		
	Spastic CP (n =10)	TD (n =10)	***F***_***1,18***_	*p *value	Effect size *d*
Utterance duration (sec/syllable)					
Mono-syllable task	0.95 ± 0.21	1.01 ± 0.06	0.858	0.367	0.213
Poly-syllable task	0.95 ± 0.30	0.97 ± 0.09	0.049	0.828	0.052
Peak vertical oral opening displacement (cm)					
Mono-syllable task	1.17 ± 0.34	1.23 ± 0.55	0.096	0.760	0.073
Poly-syllable task	1.84 ± 0.46	1.83 ± 0.68	0.001	0.970	0.009
Peak vertical oral opening velocity (mm/sec)					
Mono-syllable task	42.4 ± 10.0	42.5 ± 12.5	0.001	0.980	0.006
Poly-syllable task	73.5 ± 17.5	71.4 ± 16.9	0.079	0.782	0.066

Values are expressed as mean ± SD.

CP: cerebral palsy; TD: typical development.

The CVs of utterance duration for MS and PS tasks between groups were different (*p *≦ 0.01, Table 3). The CVs of utterance duration for MS and PS tasks of children with CP were at least three times as large as those of TD children (*p *≦ 0.01, Table 3). However, the CVs of peak oral opening displacement and velocities for MS and PS tasks did not differ between groups (Table 3).

**Table 3:** Coefficients of variation (CVs) of kinematic data in children with cerebral palsy and typical development.

Coefficients of variation (CVs)	Children groups		ANOVA		
		
	Spastic CP (n =10)	TD (n =10)	***F***_***1,18***_	*p *value	Effect size *d*
Utterance duration					
Mono-syllable task	17.87 ± 11.87	5.23 ± 2.33	10.923	0.004*	0.615
Poly-syllable task	24.30 ± 17.90	7.57 ± 4.11	8.299	0.010	0.562
Peak vertical oral opening displacement					
Mono-syllable task	24.34 ± 13.53	34.14 ± 26.16	1.108	0.307	0.241
Poly-syllable task	18.30 ± 15.82	29.42 ± 20.20	1.880	0.187	0.308
Peak vertical oral opening velocity					
Mono-syllable task	17.21 ± 14.97	24.69 ± 13.50	1.376	0.256	0.266
Poly-syllable task	15.63 ± 17.09	15.16 ± 18.78	0.003	0.954	0.014

*p < 0.01.

Values are expressed as mean ± SD.

CP: cerebral palsy; TD: typical development.

## Discussion

The present study is the first kinematic study of speech motor control in children with CP. The lack of this type of research on CP children may be due to the technical difficulty of managing movement artifacts due to head or trunk control problems in these children. In our pilot study of speech kinematic analysis, movement artifacts occurred from facial skin movement, from poor head or trunk control, and from involuntary movement in children with CP of various motor severities and subtypes (e.g. athetoid subtype). To overcome this problem, we secured subject trunk and used a specially designed facial mask. More importantly, the use of multiple measures in the current research offered an alternative to understanding the underlying abnormal motor control for speech production in CP. As the different measures used here measured different aspects of oromotor movement and speech production, they supplement each other in description of articulatory problems. The approach used in the study is likely to provide a corroborated account of the articulatory behaviors in this population.

Our study revealed that children with mild spastic CP had greater STIs in PS tasks than children with TD. In order to interpret this result, we need to understand the motor control system at the neural level, which is described in Smith [13]. To produce intelligible speech, the brain must generate motor commands to control activation of many different motor neuron pools that innervate the muscles for speech production [13]. Each coordinated movement requires temporal control and spatial control in the innervating muscles of the articulators, the larynx, and the chest wall [13]. Using Smith's model, high STI values in children with CP might reflect deficits in relative temporal and/or spatial control for speech, which might be caused by damage to the nervous system during development. Normally maturation of the neural systems underlying language processing and speech production follow a course of cortical, dendritic and synaptic development [30-32]. Damage to the immature brain in children with CP may cause variations in neural drive to muscles during speech production. As the developing system explores different solutions to achieving vocal tract goals, higher speech variability is produced [15]. This is supported by our finding that children with mild spastic CP had greater oromotor variability and relatively lower speech intelligibility in speech production than children with TD.

We also found that children with mild CP had higher STIs and relatively lower PCC in speech tasks than TD children, though the PCC did not achieve significant differences. CP group's poor performance in STI and PCC can also be interpreted at the level of oromotor control, such as spasticity, weakness, and voluntary control abnormalities. Several theories have been proposed for the pathophysiology of dysarthria in subjects with CP [33], such as weakness of speech muscles [34], abnormalities of muscle tone due either to spasticity of speech muscles [34], primitive reflexes or pathological reactions interfering the articulatory control [35], or an imbalance of positive and negative oral reactions [36]. Children with spastic CP are quantitatively less consistent in their movement output compared to TD children. STI values are related to observed differences in severity of dysarthria [37]. Thus, children with spastic CP produce relatively lower speech intelligibility in speech tasks compared with TD children.

Notably, we observed significant between-group differences in STI values for PS, but not for MS, utterances. This indicates that the STI difference between CP and TD children becomes more distinctive as task complexity increases, which is consistent with observations in several prior studies [30,38]. Previous researches have reported that children with mild spastic CP have more difficulty than normal children in processing increased articulatory demands, which is reflected in greater oromotor variability [30,38]. The utterance length and complexity on speech motor performance are related to the effects of increased processing demands on articulatory movement stability [30]. Another clinical research also revealed that syntactic complexity affects the speech motor stability of fluent speech in adults who stutter [38]. Their results suggest that complexity of linguistic structure may affect speech production processes. In our study, PS tasks place higher processing demands on articulatory movement stability than MS tasks, and therefore CP children's STI values for PS tasks were more different from TD children's.

The findings reported in the present study are of great theoretical and clinical values. First, quantitative measures such as STI and CV values are validated to be effective measures of abnormal oromotor movement in CP population in current research. Our results provide empirical data in CP children to support Smith's model [13] that describes the relationship of neural damage, muscle control and impaired speech production. Our results also suggest that deficits revealed by kinematic parameters should be considered in models of speech impairments. Secondly, the techniques and analyses used in the present study might be effective clinical tools for diagnosis and evaluation of speech motor instability. Previous works discovered that speech motor development follows a very protracted time course [39]. There is still a significant increase in consistency of oral motor coordination patterns after age 14 years [13,39]. Kinematic data might be used as indices for detecting speech motor control impairments in children with mild CP at different developmental stages. Thirdly, our results can help researchers to design treatment strategies for rehabilitating high-demanding articulatory movement, which is shown to be more challenging to CP children in this study. This type of training may be beneficial for younger children with mild CP because younger and mild damaged brains may have better neuro-plasticity than older and more damaged brains. For example, the complicated speech tasks with increased utterance length and complexity can be selected as part of the high-demanding articulatory training program. As we are uncertain whether the brain damage in children with CP can respond to such treatment, further studies are needed to investigate the treatment strategies for these children. In addition, STI index may also be used for assessing the effectiveness of treatment of such problems. Kleinow et al. reported reductions in STI in response to a speech treatment for adults with hypokinetic dysarthria associated with Parkinsonism [40]. We may potentially apply this approach to the assessment of intervention designed for children with mild CP.

It appears that the pattern of variability between mild spastic CP and TD groups is different. Children with CP had greater CVs of utterance duration for MS and PS tasks at least three times as large as those of TD children. Higher STI values and variability on utterance durations in children with CP in comparison with TD children might reflect deficits in relative spatial and/or especially temporal control (high variability on utterance durations) for speech. The temporal control indicates the appropriate timing control of muscle activations and de-activations and spatial control indicates the appropriate graded muscle activity control for speech production [13]. The deficits in spatial and temporal control may arise from poor motor coordination [13,39]. These findings may imply children with mild spastic CP may employ a different organizational unit or a different planning strategy in performing speech tasks from TD children.

The findings of this study may be limited due to its design in the aspects of sample size, measurement methods, and subject characteristics. The actual values of kinematic variables including STI, utterance durations, peak displacement and peak velocities could be influenced by multiple factors, such as instrumentation, specific tasks and signal processing. For example, the utterance durations are relatively long because the participants repeat the target syllable(s) at a relatively slow rate for clarity purpose. The STI measures varies as a function of the speech task used, and therefore it is challenging to interpret STI differences or similarities in different tasks such as MS and PS tasks. The MS task simply requires syllable repetition whereas the PS task demands distinct phonetic composition. In prior studies, STI is typically used with actual speech utterances, while the present study uses syllable repetitions as the speech motor task for open-closing oral movement in each utterance. The utterance duration was described as a kinematic event in this study. However, it is likely that a kinematic event occurred before the actual utterance by use of other articulators, which preceded kinematic detection of the lips and jaw. Besides, the tasks are relatively simple and might not sufficiently tax the motor systems of children who have minimal speech impairment. We only enrolled children with mild spastic CP and mild speech intelligibility impairment in the study. Therefore, our results can not be generalized to all cases of CP. Despite this limitation, this study has demonstrated some heuristic value relative to the dynamic organization of motor speech in children with mild forms of CP.

## Conclusion

Children with mild spastic CP showed greater STIs in PS tasks, but not in MS tasks, than children with TD do. These findings suggest that children with CP have more difficulty in processing increased articulatory demands. However, the average values of utterance duration, peak oral opening displacement and peak oral opening velocity of both speech tasks do not significantly differ between children with CP and children with TD. High STI values and high variability on utterance durations in children with CP reflect deficits in relative spatial and/or especially temporal control for speech in the CP participants compared to the TD participants. The STIs can be used as an index for sensitive detection and assessing the effectiveness in the treatment of speech motor control problems in children with mild CP.

The current research offer valuable kinematic data that support neural-motor models proposed to account for speech motor control problems. The kinematic data for speech motor control provided in this study may help clinicians to understand the speech motor control and planning treatment strategies for children with CP. This study may potentially provide directions for future linguistic and kinematic analyses in other patient populations with speech deficits. Future studies may focus on speech tasks using a variety of linguistic structures to elicit different muscle contractions and movements to provide better diagnosis and treatment for CP children at different degrees of severities and to examine the effectiveness of different treatment strategies in these children.

## Competing interests

The authors declare that they have no competing interests.

## Authors' contributions

CLC participated in the conception, study design, analysis, and draft of this manuscript. HCC participated in the experimental setup of kinematic analysis, kinematic data collection and analysis, and revising of this manuscript. WHH carried out the kinematic data collection and analysis. FGY participated in the data interpretation and the revising of this manuscript. LYY carried out the data collection and analysis. CYW carried out the data collection and interpretation. All authors read and approved the final manuscript.
